# On the Physiological and Psychological Phenomena of Dreams and Apparitions

**Published:** 1856-10-01

**Authors:** 


### Art. III.—ON THE PHYSIOLOGICAL AND PSYCHOLOGICAL PHENOMENA OF DREAMS AND APPARITIONS.

[No. II. of a Series.]

(*Continued from page* 384.)

Having offered some few general remarks on the external senses, as being suggestive of dreaming under the partially waking condition of the brain, and also when the latter is partially conscious of some atmospherical conditions, when the body is rendered more susceptible to the effects of cold and heat, that these phenomena are suggestive of trains of thought during the state of sleeping, when the dream which is thus induced, although having many discrepancies and contradictions, still preserves a kind of unity in perfect keeping with the disturbing agents which first suggested the particular association, we shall next consider the particular phenomena, when the "mental faculties are some of them in a state of excessive activity, arising from some abnormal condition of health;" premising that the trains of thoughts which are experienced by the sleeper will, in the majority of instances, still indicate his individuality, subject to certain modifications induced by the painful or other states of the body. The following instance furnishes some curious subjects for the psychological reflections and physiological phenomena:—

Partial Coma, with Faintness.—Some time since, a friend of the writer had a most singular dream. We cite the narrator's own words. He said:—"I thought myself dangerously ill, so bad that I was not surprised when the physician assured me I was dying; nay, I felt a consciousness that such was the case. My affectionate wife and dear anxious children surrounded my bed, and seemed deeply sorrowful; and when I spoke to them, they sobbed most bitterly. It was then that I reasoned with them, and pointed out to them as a source of consolation, that my condition was merely one of transition from the present existence to a better, where pain would cease, and endeavoured to impress them with the fact that this change would be for my own advantage; that I should give up a life of anxiety for one of unmixed happiness. This was urged to sooth their grief, which was intense. Then I added what seemed to me a more serious declaration: Our parting must be painful; it is so ordained; but my faith convinces me that in a few brief years there will be a reunion." He described that for a few minutes he seemed unconscious, and was partially recovered by the sobs of his family; that he attempted to resume his discourse, when his spirit appeared as if waning, and all external objects became dim and indistinct. The great problem he would soon learn; and so awfully did his position affect him, that he awoke. But he was in a state of partial *coma*, with drops of cold perspiration on his forehead, and so deadly faint that he then actually concluded that the dream was a premonitory warning "that his time was come."

In this instance it was therefore the peculiar condition of his stomach and nervous system that induced the train of thought which had so vividly affected him (as fainting is an approximation to the state of death), and this sickly sensation, and the incapacity for any kind of movement, had caused the mind to produce the scene just related, which had had all the painful vividness of a real occurrence.

How marvellous are these phenomena of the mental faculties! that as our bodily powers seem on the wane, the immortal spirit acquires a greater and more intense capacity, and by anticipation produces events which may in all probability happen at some future period.

It is, however, not our intention of multiplying examples, but only cite one instance to illustrate each separate phase under each section. Therefore, by the way of contrast, and to render it manifest that disordered conditions of stomach or derangement of other viscera, predispose to dreaming, and that the proximate cause will induce the speciality of the dream.

Mr. M—— A—— was in easy circumstances. He was a hearty man, but liable to great biliary disturbance. His temperament was nervo-lymphatic, and he was very stout in his person, and took very little active exercise. He had had in consequence several attacks of what the ancient writers called *μελαινα χολη*, with its constant results—great depression of spirits, and what we modernly designate melancholy. But under judicious regimen he had recovered a very fair state of health.

Occasionally, however, he had bilious attacks. When in this condition, he one night awoke in a state resembling insanity. He cried out in a most fearful manner, uttering the most extraordinary noises, until some of the members of his family awoke, and entered his bedroom to ascertain the cause. They found him struggling with fearful energy, striking away with his fists with great force and apparent rancour, as to render it dangerous to approach him. And this state was the more surprising, as he was a most benevolent and kindhearted man. Some of the party addressed him, and discovered that he was asleep, and evidently affected with some painful dream.

It was, however, thought to be a probable cause of his condition that the shirt-collar was too tight round his neck, and was acting as a tight ligature on the carotids, and thus impeded the circulation. By an immense effort and great presence of mind he was approached from behind, or rather at the head of the bed, and the shirt-collar was unbuttoned. It required great caution, from his enraged blows. This plan succeeded, and soon fortunately brought the sleeper relief, and he awoke much exhausted by his previous efforts; and when he recovered his consciousness he retained all the scene which had so affected him. He related that he was attacked in a most ruffianly manner by some thieves, who attempted to strangle him ; and that he distinctly remembered that he experienced a conviction that his life was in jeopardy, and he therefore made a vigorous resistance, and struck them with more than usual savagery. It was a battle for existence ; for the more he seemed to resist them, the tighter they grasped him round the throat; and in his terror he thought he must succumb, but that his only chance would be to make some one acquainted with his perilous condition. He said, with a good-tempered smile, "I roared out with all my might, 'Murder! murder!'" We have mentioned this was done so lustily as to arouse the sleepers in an adjoining room. It is evident that the tightness of the shirt-collar, induced in all probability by his restless condition, by causing him to slide downwards from the pillows, had occasioned the annoying circumstance; and in this instance the dream may have saved him an apoplectic fit; for, with the constriction round his throat, in a comparatively short time such a casualty might result.

Here is another example, that the state of the body, even when there is not any indisposition, may predispose the particular kind of dream. And the one we select from many others, arises from a striking phenomenon to be indicated. A friend of mine awoke one morning coughing most violently, which excited in him expressions of anger, as each involuntary spasmodic effort proved unavailing in relieving him; he said in a most pettish manner—"Why do you hold that lucifer match so near me? It's choking me!" His wife, who was lying awake, assured him that there had not been any lucifer match ignited. This, for a moment, rendered him still more irritable, and he coughed with greater violence. And yet he was, evidently, not exactly awake; and when this extreme effort relieved him from the phlegm, he recovered his perfect consciousness, and had not the slightest remembrance of his harsh expressions, and the angry tone in which he had previously uttered his protest, for he said, "How curious it is, that I have just dreamt that one of the children had used a lucifer match quite under my nose, although I told him that it annoyed me; and, the fact seemed that instead of heeding my reproof, he repeated the annoying experiment." Now it was evident that the irritation resulted from an accumulation of foreign matter, which gave the tendency to cough, and the supposed burning sulphur might appear a myth of the partially awakened faculties. But as he was a man of accurate observation, he himself gave a reason for the supposed offensive combustible, for, strange to say, the dreamer was an habitual smoker, having a cigar almost constantly in his mouth. Having a slight cold on his chest, had caused an accumulated deposition on the mucous surfaces, which had, in consequence of his habit, received an empyreumatic savour, and owing to this circumstance was the suggested notion of the supposed cause of his sense of suffocation.

We have a number of notes of dreams induced by some local pain, either from a certain active inflammation or from some position which may produce a similar sensation. A lady of a highly sensitive temperament, with great susceptibility of both mind and body, and with such strong moral perceptions, that she was one of those beings whom nature designed as a Sister of Mercy, had, on one occasion, a most painful dream. She felt greatly distressed because there was a most extraordinary epidemic in the town, which curiously attacked the sufferers on the bridge of the nose, and soon destroyed this important feature. The agony she endured from seeing so many noseless persons affected her greatly, and from her extreme sympathy at such a melancholy occurrence, she awoke; but for some time she could not disabuse her mind as to the reality of her painful vision; as she gradually gained more complete consciousness, she ascertained that her own hand rested on her nose, and had affected temporarily the circulation, occasioning her much pain in the nasal organ. This circumstance had evidently been the predisposing cause of the dream. These instances differ in many particulars from what is called, by Plato, *Ephialtes*, or nightmare; and they are often classed under this section. But in true *incubus*, or "nightmare," the respiration is seriously affected, and in consequence of some disturbed action of the heart, either mediately or immediately, there are always associations of immovability on the part of the dreamer. The mediate predisposing cause is pressure on the heart, by some uncomfortable position; the immediate tendency arises from gastric disturbance, when the pneumogastric nerve, by reflex action, affects more or less the heart's action.

INCUBUS, OR NIGHTMARE.—From the experience of many observers, and my own, it seems evident that in all cases of *incubus*, a disturbance of the circulation is the predisposing cause, and the dreamer thus affected invariably seems to lose all power over the voluntary muscles, and this condition of the muscular system differs from others to be subsequently indicated.

And, further, we make remark, that in true incubus the intercostal muscles are implicated, hence the impotent efforts of the dreamer to resist attacks, and so forth. One example will suffice to illustrate the latter statement. A gentleman of our acquaintance, of a robust and active temperament, and well-formed head, dreamt that he saw a low, dirty-looking boy open his bed-room door, and in the most impudent manner stare him in the face, seemingly without heeding that he was wide awake; that from this circumstance he became alarmed, from a conviction that there were some adult associates at the outside of the bed-room; that he attempted, nevertheless, to speak to the intruder, but he could not, and yet he saw, with a sense of indignation, the juvenile thief open different drawers, from which he abstracted a gold watch, diamond studs and rings, with a handful of notes and a bag of sovereigns; and after packing them up deliberately, the delinquent came up to his bed-side, and with a most impudent leer, nodded his head, and said, "Good night, old chap." The wrath of the sleeper was so great that he tried hard to rise and seize the thief, but could not; he was equally impotent in the attempt to throw something at him, or to make any noise to arouse his servants. But these efforts awoke him, lying on his left side, and his arm pressed against the heart, whilst his lower extremities were cold. We may, therefore, reasonably refer the whole phenomena to the fact, that some of the muscles were deprived of a due supply of blood, and to an excessive supply of this fluid to the brain.

An amusing instance of nightmare occurred to a relation of the writer, induced by actual pressure on the chest. We will relate it in his own words:—

CASE OF NIGHTMARE FROM ACTUAL PRESSURE ON THE

Chest.—"I had been," says the narrator, "travelling all day on the outside of a coach, and as the weather was cold, had imbibed a larger quantity of brandy than is my usual custom. It was late at night when the coach arrived at the inn where I had proposed sleeping; and after taking a hurried supper and a glass of brandy-and-water, proceeded to my dormitory. That illustrious official, 'the Boots,' had just retired to bed, and so slippers were a luxury I had to dispense with; and as it happened, this was a fortunate incident.

"The supper and grog made me somewhat feverish, and soon I felt a stupor coming over me, and fell into that kind of sleep when one is not altogether oblivious of our own sensations. In this condition I had a most painful dream. It seemed that the coach by which a large party were travelling was upset, and all were thrown helter-skelter; but that one of them fell on me with such force, that it seemed probable, that although my life had been spared in this accident, that it was my fate to be smothered by a huge mass of human flesh, which appeared to press to such a degree, that I felt, if unrelieved, that I must soon 'give up the ghost.' I therefore entreated this torturer to remove himself, or he would soon be the death of me, and much oppressed, my indignation was in a furor, as he seemed to be laughing at my sufferings. Then I made every effort to throw him off, and so great was this effort, that it awoke me.*

"You may judge my surprise, that there was, even to my waking sensations, an actual weight on my chest; and as I breathed, it seemed also to breathe. In the state of my health, and the disturbed condition of the mental faculties (owing to the stimulation of the previous day), it was my veritable belief that I was dying, or else it was some curious condition of fever, so certain it was that this pressure was real.

"Then I listened, and tried to ascertain if it were a reality or a delusion, and had just decided that it must be 'night-mare,' when, instead of a 'dead weight,' the pressing tormentor slightly moved its position higher and nearer to my mouth. This actually terrified me, although it was years before *burking* had been rendered a practical art. There seemed no time to be lost; so, arousing all my energies, I bellowed out, in no very pious strain, 'What the d—l are you?' Instantly something bounced off my body on the floor, which was ponderable evidence that the oppression had not been anything illusive, and I came to the conclusion that it must be a cat,—an animal, by the way, that I feared more than hated. My first plan of operation was to open the

* It may be worth noticing, that when the dreamer's sufferings are so intense, that the continuance might affect his sanity, he invariably awakes to a state of more or less perfect consciousness.—Author's Note.

door, to give the intruder a chance of retreat, and stood with one of my heavy boots in my hand, armed for an onslaught, when a big black tom cat rushed forward, but not before my missile was hurled with such force, that if it did not touch 'Tommy,' it did the door, with so much noise as to disturb the slumbers of the occupants in the dormitory along the whole landing. But as I had gently closed the door, many were the conjectures of the source of the disturbance; and which the next morning, in justice to the company, I explained."

There are other forms of dreaming, in which the muscular system is implicated, either as a cause, or as the servant of the brain and nervous system. The following instance, narrated to us by a gentleman of great talent and philosophical tendency, is now published to elucidate a passive condition of the muscles without any marked effect of disturbed circulation, as in true *incubus*. Its *psychical* character is similar to hundreds of others, furnishing another proof of the difficulty of tracing with any marked accuracy the minutiæ which form the varied circumstances of any particular dream. Our friend writes—

"One day I saw two boys in the street, one of them much older than the other. The elder was teasing the junior, who said, in a crying tone, 'It's very cruel, Johnny, to treat me in such a naughty manner; I'll tell father!' I interfered, and chided the tormentor, telling him that his conduct was cowardly and unfeeling, and thus for a time saved the little fellow from any further persecution; for I watched their movements, and observed the elder with his arm placed in an affectionate manner round his brother's neck.

"That same night these meagre materials furnished all the incidents of a dream, which has occasioned some curious matter for reflection to myself and to others to whom the details have been related. I was musing in a field where a number of boys were playing, and watched their gambols with extreme pleasure. But soon two boys near the spot where I stood attracted my attention, as the younger was blind. The poor little fellow complained that he was very thirsty, and he asked his elder brother to take him to the tank on the opposite side of the meadow. He refused, in a rather angry manner; but although the colour came into the face of the bereaved little fellow, he uttered not a word of complaint, only repeated his request in a most urgent and more importunate manner. But his wishes were unheeded. As I had seen this tank, which was constantly filled, being supplied by an under-ground spring, so that the clear, bright, cold water constantly flowed from its sides, and being very sorrowful that the blind boy should suffer from thirst, I proposed to guide him, but could not move. My limbs were almost instantaneously paralysed. 'Poor little fellow,' said I, 'what will you do?' 'Go myself, sir; I can find the way.'

"And no sooner said than done; for off he ran towards the tank of water, and I trembled lest he should fall in and be drowned. As he took the exact direction to it, without stopping, my anxiety was painful, so that to myself I appeared to be terror-stricken, as I could not move so as to render him any assistance. At length, to my great surprise and relief, he stopped suddenly when within three or four yards from the reservoir. Then he seemed to listen, as if to judge of its distance by the gurgling sounds of the water as it rolled over the sides on the pebbly bed beneath. Having apparently satisfied himself, he suddenly dropped on his knees, and in that laborious way he gradually approached the precious beverage; when it was within his reach, he was bending his head, and drank from the precious stream, and so greatly moved he seemed to be at the comfort he derived from the refreshing beverage, that his own tears flowed copiously, and became so sympathetic, that I awoke."

Our friend said, that he was lying on his back, as easy as possible. There was not any pressure on the heart, there was not any cramping of the limbs, and yet he felt the same incapacity to move his body, as if he had been actually terrified by some sense of imminent danger, or by some marvellous event. The effect only continued for a short time, and his normal volition was restored.

It is certainly a curious case, and we have only one solution for the phenomenon. Taking our *data* from what occurs in the waking state, it may be remarked as a matter of indisputable experience, that terror tends to paralyse the muscular powers. And even in states of ordinary fear, they are rendered feeble in their action, so as to make even a strong man totter. We may, therefore, assume some little difference in the events narrated, that is so far as to their order of occurrence; and that in the first instance our dreamer's *caution* was excited, and hence in his dream, it had the same paralysing power as would have been experienced in the hour of consciousness.

Dugald Stewart thinks, "that in dreaming we are influenced by the laws of association, as we are in the waking state." "The mind," he thinks, "resembles 'the poetical power;' the power of combining and mixing the most heterogeneous circumstances; and thus fashion the most absurd, grotesque, and monstrous combinations." We quote from memory. But these are the views this philosopher propounds.

In our own investigations of the phenomena connected with dreams, we find that the nocturnal vision, which is influenced "by the laws of association," is only when that *dream* has any reference to recent events or occurrences. That these links ot memory may have been formed by reading or conversation ; and even then the circumstances reproduced are somewhat modified, and the *dramatis personæ* are changed, the principal part being sustained by the dreamer himself.* Before we cite any evidence, we must offer a few remarks on those mental powers which perform a part of the intellectual process principally concerned in reproducing events and scenes which have occurred. We speak of them as perceptive faculties, and their functions, although various, are all essential for the appreciation of all objects of which the external senses can take cognizance. By these powers we take cognizance of individual entities, note their forms, proportions, colours, densities, and indicate their particular functions, their relative numbers, duration, and so forth. It is important for our purpose even to note thus briefly their respective functions. For during the dreamy state, these perceptive powers are often involuntarily excited. That is, they reproduce past impressions when the original objects are no longer present ; and so vividly they appear, that during the vision they have all the same influence in producing pleasure or pain which the things or events in their material reality had done. But the congruous or incongruous scenes which may be presented during our nightly visions, will depend on their being natural and consistent, or disjointed and extravagant, by the accidental circumstance whether one or many of the perceptive faculties are associated in these reminiscences. If there are similar combinations, as in the waking hours, nothing will appear either incongruous or extravagant. If such is not the case, then, indeed, the dream is a mere "fancy sketch." In the mysteries of our being, we find that in dreams there are often past events resuscitated, sometimes with a faithful vividness, but at others commingled and without any definite associations. Yet we think, as a general view, that these unrefreshed and active perceptive faculties more frequently take cognizance of recent scenes. We will cite one of our own from the phenomena subsequently noticed, being interesting in a physiological point of view.

CURIOUS REMINISCENCE DISTORTED.—A friend of the writer, Mr. C. W. Day (author of "Hints on Etiquette," &c., &c.), on his return from one of his journeys in Switzerland, published in one of the monthly magazines a brief account of his perils and adventures. He particularly described an exciting incident at the *Mer de Glace*, in which there is a deep fissure, (the mass of ice having split on some occasion,) but over this gap the traveller

* This egoism of the dreamer we shall have to refer to as we proceed with our subject.

must spring, or suffer the inconvenience of retracing his journey. And Mr. Day, in his narrative, rendered it highly exciting and graphic. We could see him, so to speak, standing on one of the slippery surfaces of this "sea of ice," and with daring courage hazarding a leap over the gulph, with all the danger of making a footing on the opposite slippery resting-place. The account was very artistic, without being highly coloured, but strictly "true to nature," although slightly idealized.

After reading the very able article, and conversing with the author on some of the details, I became so deeply interested, that on that same night, while in bed, I thought repeatedly of the exciting adventure, until I fell into a rather sound sleep, and dreamt that I was travelling in a foreign land, and was on the confines of an enormous large forest, which seemed to stretch for many miles, even as far as the eye could reach. The evening sun had sunk beneath the distant mountains, and all appeared most beautiful, yet a sense of melancholy oppressed me; for within the forest could be heard a discordant chorus of wild beasts, roaring, growling, groaning, and all seemed as if approaching the spot where I stood; such was my unpropitious position, so far as retracing my weary steps, the attempt could not be made without danger; yet before I could gain the open country, it was necessary to pass a broad, deep ravine,—the waters in it echoed for some time after a stone was thrown into it—this also was like a sepulchral voice, announcing its readiness to receive me as another victim. Over this ravine, with its sunken sea, there was a rude bridge, constructed of deal planks, dovetailed together, and it had no hand-rail to protect a passenger. I have a distinct recollection that in my dream I reasoned thus—If I remain, there was every probability of being torn to pieces by some wild beasts; or if an attempt were made to cross the "bridge of planks," and dizziness overtook me, that death in that case would also be inevitable; still the latter seemed most preferable of the two possible accidents. When I had decided, and had gained the centre plank, I lost my balance and fell, but in so doing I made a desperate effort to grasp the bridge, and succeeded in doing so; the whole weight of my body depending on my left arm, the pain of which was so great that I groaned audibly, and to my great relief my wife awoke me, asking me, "What is the matter?" I replied, Thank God, it is but a dream, but what a fearful one. There were not any symptoms of *incubus*, as in the latter state volition over the voluntary muscles is lost, even to the seeming consciousness of the dreamer. But in this case, there was this phenomenon—I had in my dream exerted the muscles of my arm, suspended by them alone, and had evidently used the same amount of effort as if it had been an actual event, for the left arm ached to such a degree that it was evident the muscular force, having been directed by a similar act of volition, would have been sufficient to sustain the whole weight of my body even, for a brief period. Nor did the painful sensation cease for two days.*

We are also tempted to give a case of muscular lassitude, induced during sleep, by showing that long-sustained muscular exercise, even in a dream, may bring a similar amount of fatigue, as when actively performing an equal amount of labour when in the waking state. This, we apprehend, results from the fact, that the muscular force put into requisition requires a similar amount of nervous power as if actively exercised, and that there is a corresponding demand made on the brain and spinal nerves for this purpose.

Mr. L——, a very superior musical artiste, who was an organist of some celebrity at the ———, told us an interesting dream which had occurred to him, and of which, from its effects being so marvellous, he asked us to explain the phenomena. He said that he had been practising, with great zeal and labour, some of Sebastian Bach's most elaborate fugues, until he had acquired the most facile execution, even with the most elaborate; and that he continued these exercises, from finding the highest emotional gratification from them. One night, after his usual daily occupations of teaching music, he went to bed, but he did not recollect whether he had felt more than ordinarily fatigued. He dreamt that he had to play these fugues before a very large congregation, but he found, to his horror, that the pedals would not move, and that it was utterly impossible to give any proper effect to these sublime compositions—that he tried to do so with great and intense anxiety, and with the most indomitable perseverance; but the difficulties increased, and his chagrin and disappointment were great, as he had never anticipated the possibility of such a complete failure. Hence, he added, that he made still greater efforts, trying with all his energy and might to make the pedals act; but with all his additional labour he could not succeed, and under a sensation of despair, he awoke. He said that he was quite jaded, and physically prostrated, particularly his legs and arms, which were not only tired, but they actually pained him, just in the same degree as if his dreamy adventure had been an actual reality.

There is not a doubt that if he had not actually used the muscles of his arms and feet, that he had expended a similar amount of nervous power as if the muscles of both the legs and

* We shall have some few further remarks to make on this subject in the conclusion.

arms had been exercised under similar circumstances, whilst under the perfect volition of consciousness.

Every physiologist will at once admit that in the latter instances there is a marked difference in the condition of the muscular system in NIGHTMARE.

We are tempted to narrate another instance to prove that precisely a similar class of phenomena were observed—namely, that the state of the muscles in this dream was similar, or nearly so, as if the occurrence had been real, and not a vision of the sleep.

A gentleman who practised as a surgeon told us that he dreamt that he was sent for by one of his patients to go and see a poor fellow who had broken his leg, and that when he went into the house the poor creature was groaning from excess of pain. The limb was much swollen, and red, whilst a portion of the bone was visible on the surface. Some "village bone-setter" had tried to reduce the fracture; but from his great ignorance, he had only tortured the sufferer. The family were in a state of great distress; and altogether, he said, that all his higher motives were urgent to relieve the patient and comfort those around him. He distinctly recollects that he took his coat off, and, after great efforts, he succeeded in bringing the bones in juxtaposition. It was a labour rendered more difficult by the sensitive state of the injured leg and highly nervous condition of the invalid; so that, what with his screaming and groaning and occasional movement and resistance, at the same time roaring out, it was greater torture than he could bear; our surgeon says, he had a repetition of his efforts, but he felt fatigued and qualmish, which sensations awoke him. To some questions we put to him, he declared that his arms were even more tired than if he had actually been performing the same operation; and he also thought that the positive muscular effort he had been making was similar in its waste of power and consequent exhaustion.

Having entered on this inquiry with the view of unfolding the phenomena of dreams, and with the hope that the views submitted would have some practical advantage, we shall proceed with our subject, and shall next consider the many anomalous forms which are experienced by the dreamer when under the influence of narcotics or alcoholic compounds.*

* The writer of this Essay has tried experiments on himself, with the view of ascertaining the specific effects of narcotics (opium, laudanum, morphia, smoking strong tobacco, &c.), and he has noticed that the dreams resulting from them are either grave or gay, the perceptive faculties being active, and the sense of the beautiful or the marvellous acting with them. In other words, *narcotics* act principally on the *cerebrum*, whilst alcoholic compounds stimulate the *cerebellum* and base of the brain, and generally induce animal and prurient associations in these visions of sleep.—AUTHOR'S NOTE.

We may incidentally remark that NARCOTICS produce a species of delirium, and then, indeed, the dream may resemble some form of insanity. The one now to be submitted is, indeed, curious, the details of which were furnished by the dreamer, who writes thus:—"During the time of my visit to Scarborough in 1827, a course of lectures was delivered on the science of phrenology, which were illustrated with a great number of *crania*. One night, a medical gentleman and a friend of mine, who, like myself, was a visitor at this beautiful watering-place, went early to have some conversation with the lecturer, and whom he asked to give him an account of the personal history of some of the skulls on the table. He and I were much amused at the condensed biographies which were given of this motley union of philosophers, thieves, murderers, philanthropists, &c.; and the idea occurred to me that if these skulls could utter an address, some of them would be indignant with being placed with so many disreputable associates.

"When the lecture concluded, we proceeded to our hotel; and feeling much fatigued and jaded by the continued tension of my mind, I was induced to smoke some strong cigars, to act as a sedative. When I went to bed, I had a vivid perception of the lecturer's collection of skulls, and pondered over the important reflections he had deduced from their respective organizations. Whilst these ideas still engrossed my attention, I fell asleep. But the train of thought must have continued; for it seemed to me that I was tempted to go and examine them again, and whilst doing so, the skulls became suddenly animated, and each was immediately attached to a fleshless skeleton. And then an extraordinary scene took place. Each seemed to manifest all the excessive passions which had formerly distinguished them, except the philosophers, who remained calmly contemplating these furious beings. A sense of agony came over me; for either they mistook me for the lecturer, or else their ire was roused for my having been such a Paul Pry to have dared to inquire into their respective histories; and although it seemed quite awful to observe this 'dance of death,' yet it excited my risible faculties. The skeletons then rushed towards me, and in my extreme terror I awoke: my hands were cold, and my skin clammy, like when faint from the effects of the nicotine of tobacco in some states of the stomach."

In this dream the perceptive faculties, with *wit*, *ideality*, and *marvellousness*, were under simultaneous excitement; whilst the reasoning powers must have been in the inactive state of profound sleep. If the latter had not been the case, the absurdity of fleshless skeletons bending their fists, and running and jumping in such a wild manner, would have been an obvious impossibility, as they could neither move their arms nor legs when there did not exist any muscles, but merely the bony framework of these skeletons.

If there are "sermons in stones," there are moral inferences to be drawn from dreams. We are tempted to give the following, and vouch for the accuracy and correctness of the report. It is a curious political dream, in which there is detailed the trial, sentence, and execution of the dreamer.

Before, however, giving the details, we must premise that the gentleman who narrated it to us, and with whom we are intimately acquainted, had taken an active part in politics, when it was unsafe and injudicious to express any extreme liberal opinions. It was at the time when Lord Sidmouth and his colleagues suspended the *Habeas Corpus* act, about the period of the trial of Wooller, the editor of the "Black Dwarf." Our friend was enamoured with his lengthy and able defence, and had a particular desire to be himself a political martyr. This arose from the fact that he had himself a well-stored mind and a great capacity for public speaking, of which, by the way, he was most vain. And, although he would have shrunk from the commission of any crime, yet, influenced by a powerful *love of approbation*, he did not consider the being arraigned for political opinions to be a violation of moral rectitude, or any infringement of his duties as a citizen. He had but the one idea, that of making a display of his stores of historical facts, and of making an eloquent appeal on the right of an individual to advocate such views as in his judgment would tend to elevate the human family. These thoughts floated in his brain, and whenever he had an opportunity, in public or private, he would descant on them with great ardour. The meetings he attended were held at the parlours or club-rooms of public-houses, in which clouds of smoke enveloped the assembly; and although he was not much of a drinker, he indulged excessively in "the Indian weed," so that he might be considered always narcotised. We have been forced to give these phases of the mind of our "dreamer," and which will enable us to trace the train of ideas in his sleeping vision, and to account for its curious results. He had been for some hours in a dense fog of tobacco-smoke, when he returned to his lodgings, tired and stupified by the poisonous atmosphere, and quickly retired to his bed. He dreamt that he was arrested for some speech on his ultra-radical views of government, in which he had enforced his favourite principle—"that men were not under any obligation to obey bad laws, any more than that a child could be considered guilty of disobedience to a father who might urge him to commit an act of moral turpitude." He says that he vividly remembers being committed to prison, and that his trial took place after many weeks of incarceration, when he made a most eloquent defence to a crowded court; but the jury pronounced a verdict of guilty. He heard a murmur throughout the court, which was soon suppressed. Then the judge put on a black cap, and pronounced the awful sentence—that he must suffer the penalty of death by hanging, for his treasonable conduct. Soon his busy and disturbed mind produced another exciting scene: he was brought from the gaol, pinioned and guarded, and had to mount the platform. He says that he distinctly felt the rope round his neck—heard the bolt withdrawn—and was conscious that he was at that moment launched into eternity! That is, considered himself dead, and seemed quite aware that he was put into a shell to be buried, and yet all that he actually experienced was a different state of his feelings, which, even in his dream, was to him a psychological puzzle; for he distinctly heard the comments made on his character, and many eulogiums passed on the very speech for which he had suffered the penalties of the law. He distinctly heard people defend the soundness of his principles and the honesty of his whole life; and he felt a peculiar satisfaction, verifying "the ruling passion," in both the waking state and his dreams, was a strong vanity. He added, that those who carried his coffin began to move, and that they once stumbled; and a strong notion affected him that they would let his pent-up house fall, which he thought would disgrace his corpse; and this fear awoke him to a state of mental consciousness, when he found that the collar of his night-shirt had slipt on one side, and not acted as a tight ligature to the throat, but that the button pressed on the jugular vein. The pain of the latter, and the obstruction to the venous blood, predisposed the whole of the phenomena of his remarkable dream, in which there was a renewal of many of the vagaries of his waking thoughts. The dream, however, left so strong an impression on his mind as to modify his opinions, and induced him to give his attention to moral science, and to eschew party politics.

We may incidentally remark, that the *egoism* of the dreamer not only insures his individuality, but as a consequence renders him invariably the hero of every incident, or else the principal actor. If there is a fight, he is dealing his blows with fearful effect and deadly consequences to his antagonist. If a lecture is given on any subject it is by himself, or else he is propounding its errors or unsoundness. Should he dream of a riot, he suppresses it. It was his eloquence which acted "like oil on the troubled waters," and stilled the commotion induced by the passions of the people. Even when there occurs, during the nightly vision, any great inconsistency, or obvious discrepancy, it still seems correct, as in the dream it is his own mind which furnishes every associated circumstance.

It is worthy of a passing notice, that often there is a great craving among the insane for tobacco or snuff, as if they had an intuitive perception of their sedative tendencies, and hoped by their aid to allay their morbid irritability. And yet if these narcotics act on the disordered brain as they generally do on a sane person, we have the most indubitable evidence, from a vast number of smokers, that their dreams are not only most vivid, but often the most painfully distressing, from their apparent naturalness. Part of which effect, that which is depressing to the dreamer, may be occasioned by some gastric disturbance, as few inveterate smokers escape this penalty.

We were somewhat dubious where to place in our collection the following dream, for it was evidently suggested by the state of the weather:—

Mr. T——, a rich merchant, by birth a German, was rather out of health, and was recommended by his physician to spend a few weeks at Brighton. He was, like most of his countrymen, a smoker, scarcely being ever without a cigar, unless at his meals; but whether from this habit, or the constant activity of his mind, from his extensive mercantile transactions, he was highly nervous, particularly if the weather was cold, so as to affect the cutaneous circulation. On the occasion we have to speak of, the weather was so boisterous, that when he walked before dinner on the cliff, his hat blew off, but he recovered it. As he was obliged to remain in his apartments, and being too listless to read, he puffed away in a more than ordinary degree, and about ten o'clock went to bed, but not "to sleep;" at least, from the wind shaking the house, he was in bodily fear that the roof would be blown off, or some other casualty. After tossing about in a restless manner for some time, he fell into a profound slumber; but the angry gusts still rattling the windows, which seemed every now and then as if they would be staved in, he must have partially appreciated. For he dreamt that he was at a foreign port, and had gone on board of a ship homeward bound. The weather was stormy, and the wind rattled the sails in a most fearful manner. Some expressed a desire to return to shore, but the captain heeded not their wishes, as it would be incurring danger. The sailors were shouting and running about the deck, all bustle and confusion, from the rapid manner they had to attend to their multiform duties. At length the vessel was fairly out at sea, which ran mountains high, every now and then coming over their frail bark like an avalanche, which made the landsmen fancy must sink it. Our dreamer was at first terrified, but ultimately he conversed with the crew and the different passengers, and examined the luggage to ascertain if all his own were right, when he found that he had left his hat-box, and that to his surprise he had been all day without any covering to his head!

After some time, the vessel arrived in port. The custom-house officers overhauled the trunks and bales, when Mr. T—— remembered some packets locked up in the captain's cabin, and among them his hat-case. But strange enough, when he has the keys given him, he attempts to descend to the cabin, the motion and rocking of the vessel prevent him; he is angry, and awakes. When indeed, he says, the storm had so increased, that he felt his bed shake.

Besides the state of the weather, the hat blown off on the cliff, and the sea being rougher than usual, were all the materials to work up the dream. But in this instance, and innumerable others, the mind fashioned a vessel, created different men—with their individualities of character—gave them certain ideas in common with their respective class, (as for instance, the captain, seamen, passengers, and custom-house officers), and supplied them not only with thoughts and opinions suitable to each, but also the very words with which they expressed themselves.

As a contrast to this matter-of-fact subject of a dream, we are tempted to cite one where the *ideality* of the dreamer gave a rather *outré* train of thought, which had had some positive *data* curiously distorted.

It is, indeed, an old and a trite remark, "that very often dreams are but the continuation of our waking thoughts," which, however, may be mixed up with confused reminiscences, and so modified by a want of unity, time, and place, that on comparing them we have some difficulty of tracing any resemblance between the waking and the dreamy thoughts. There is, however, a moral in the one we propose relating. A learned friend of ours writes thus:—"Some time since I had a visitor, a vain young man, who talked in a rapid way, and fatigued me as much by his manner as by the heterogeneous mixture of all kinds of subjects on which it pleased him to discourse. He touched on one, then changed to another, with the rapidity of a fly gliding over a fluid; then for a time he dwelt with rhapsody on some dish of which he had partaken, or some scene he had witnessed. Next he speculated on political and moral science—talked of education, beautiful women, war, machinery, and murders. Thus he rattled on from subject to subject without either order, arrangement, or connexion. Tired with the effort to keep my attention, or to follow him in this mental race, which was so tortuous, I was glad to avail myself of the conventional privilege of a convalescent by retiring to bed, where I soon fell into an uneasy sleep. Then I dreamed that I stood by a very large transparent reservoir, which was filled with water or some other clear kind of fluid, the upper part of which was like a polished crystal. Wondering what could be the use of this reservoir, I remained fixedly gazing at it. Soon there appeared to rise up from the lower portion a vast number of objects, which floated about with rapid motions until they reached the surface, when they passed over the sides or the upper end, and disappeared. 'This,' I exclaimed, 'is a fluid pandora, a magical reservoir!' My curiosity was excited, and I continued to observe the operations with the greatest attention. Sometimes the turbid matter at the bottom of the vessel became greatly agitated, and immediately flowers of various shapes and colours floated gracefully for a brief time, and then vanished. These flowers were succeeded by a vast number of animalcules of different sizes and forms; and they also passed away before my astonished senses. Various grim and bleeding victims then rose up, showing their 'raw heads and bloody bones;' and after harrowing my feelings for a few minutes, disappeared in 'mid air,' followed by fragments of machinery, nondescript animals, portions of pictorial and graphic art, which all equally mocked my gaze; and as the vain attempt was made to endeavour to compare them with what I had seen, they all exploded into a thin and impalpable mist!

"Such was this magical dream, so full of marvellous legerdemain, that my very eagerness to comprehend their meaning awoke me. With my first imperfect consciousness, this vision seemed 'strange, passing strange,' and seemed to intimate 'that the cloud-capped towers' and all earthly things were about to dissolve, and not leave a wreck behind; for from the intense headache I endured, it had not occurred to my mind the fact that all this confusion and the different mutations had been fashioned from the still Babel of subjects passed before my mind's eye by the compounds of small-talk of my visitor!

"If, therefore, my mind had not been impressed with the injudiciousness of talking in a lax manner on every subject, this dream would have pointed a moral, if it could not adorn a tale; and hence may be useful in any investigation of the philosophy of dreams."

Our friend afterwards said, that so vivid had been the brain-conjuring of his dream, that he had some difficulty of disabusing his mind as to the reality or not of the incidents.

(*To be continued.*)

